# Pathological count of IgG4-positive plasmacytes suggests extraophthalmic involvement and relapse in patients with IgG4-related ophthalmic disease: a retrospective study

**DOI:** 10.1186/s13075-022-02757-2

**Published:** 2022-04-01

**Authors:** Yiqun Yuan, Fengxi Meng, Hui Ren, Han Yue, Kang Xue, Rui Zhang

**Affiliations:** 1 Department of Ophthalmology, Eye & ENT Hospital of Fudan University, Shanghai, 200031 China; 2 Laboratory of Myopia, Chinese Academy of Medical Sciences, Shanghai, China; 3 NHC Key Laboratory of Myopia, Fudan University, Shanghai, China

**Keywords:** IgG4-related ophthalmic disease, Pathological findings, Serum IgG4 level, Systemic involvement, Relapse risk

## Abstract

**Background:**

IgG4-related ophthalmic disease (IgG4ROD) is a phenotype of IgG4-related disease (IgG4RD) with ophthalmic involvement. The pathological IgG4+ plasmacyte count has only been used for diagnosis. We aimed to explore its possible clinical value in the management of IgG4ROD.

**Methods:**

Fifty-five pathologically diagnosed IgG4ROD patients were included, and their clinical, pathological, serological, and radiological findings and treatment outcomes were reviewed and analyzed. The pathological IgG4+ plasmacyte counts in lesions from different anatomic sites were compared, and their association with serum IgG4 concentrations, systemic involvement, and relapse risk was analyzed.

**Results:**

The patients were divided into groups according to the anatomic site of their biopsied lesions, namely, the lacrimal gland, extraocular muscle, and orbital soft tissue. No significant difference was found in the pathological IgG4+ plasma cell counts among these groups (*p* = 0.975). The pathological IgG4+ plasmacyte count positively correlated with the IgG4 concentration in peripheral blood (*R*^2^ = 0.5469, *p* < 0.001). The serum IgG4 concentration and the pathological infiltrating IgG4+ plasmacyte count were significantly higher in patients with extraophthalmic involvement (*p* < 0.001 and *p* = 0.005, respectively). The areas under the receiver operating characteristic (ROC) curve (AUCs) of the serum IgG4 level and pathological IgG4+ plasmacyte count for identifying systemic involvement were 0.897 (*p* < 0.001) and 0.759 (*p* = 0.015), respectively. The patients with relapse had higher levels of serum IgG4, more germinal centers (GCs), and infiltrating IgG4+ plasmacytes in lesions. Multivariate Cox regression analysis revealed that a pathological IgG4+ plasmacyte count of > 150/high-power field (HPF) and an elevated serum IgG4 level of > 500 mg/dL were risk factors for relapse after steroid treatment.

**Conclusions:**

Lesions from different ophthalmic sites in IgG4ROD patients have similar counts of IgG4+ and IgG+ plasmacytes. The quantity of pathological IgG4+ plasmacytes corresponded to the serum IgG4 concentration in patients with IgG4ROD and could be meaningful in identifying systemic involvement and predicting subsequent relapse.

## Background

IgG4-related ophthalmic disease (IgG4ROD) is a recently recognized condition characterized by tumefacient ophthalmic lesions infiltrated by IgG4-positive (IgG4+) plasmacytes [1]. The histopathological feature of dense IgG4+ plasmacyte infiltration is the most essential hallmark for diagnosing this condition [2, 3]. IgG4ROD shares similarities with other inflammatory diseases referred to as IgG4-related diseases (IgG4RDs). A significant proportion of patients with IgG4ROD have systemic disorders such as sialadenitis, thyroiditis, parotitis, and retroperitoneal fibrosis [4]. In addition, most IgG4ROD patients show elevated concentrations of IgG4 antibody in peripheral blood. Although the disease responds well to corticosteroids, nearly 50% of patients experience recurrence after steroid treatment [5].

Accumulating studies of IgG4RD have demonstrated the usefulness of serum IgG4 levels in evaluating dynamic disease severity and monitoring relapse [6–9]. Autoimmune pancreatitis (AIP) patients with elevated serum IgG4 concentrations have been reported to have a higher incidence of severe symptoms and extrapancreatic lesions [10]. A prospective IgG4RD cohort study in the UK suggested that a serum IgG4 level of ≥ 280 mg/dL at diagnosis is useful for predicting multiorgan involvement and the risk of relapse [11]. The relationship between the numbers of IgG4+ plasma cells and serum IgG4 concentrations remains obscure, although several previous studies have noticed that the counts of IgG4+ plasma cells tend to be higher in patients with AIP and other non-IgG4-related diseases and higher IgG4 concentrations [12, 13]. The count of IgG4+ plasmacytes infiltrating lesions has only been applied to diagnosis.

IgG4ROD describes IgG4RDs that occur in ophthalmic sites, including the lacrimal glands, trigeminal nerves, extraocular muscles and other orbital soft tissues [14]. The histogenesis and histologic characteristics vary in different ophthalmic sites. Before investigating the clinical value of pathological IgG4+ plasmacytes, we noticed that the numbers of IgG4+ cells infiltrating different lesions varied from organ to organ [15]. Quite different cutoff numbers of IgG4+ plasmacytes were applied to organ-specific IgG4RD diagnosis criteria. It has not been determined whether the counts of IgG4+ plasma cells in different ophthalmic sites are similar. In this study, we calculated the pathological counts of IgG4+ plasma cells and investigated their potential role in the management of IgG4ROD.

## Methods

### Patients and tissue samples

This study was a retrospective review. Consecutive patients with a diagnosis of IgG4ROD referred to the Eye and ENT Hospital of Fudan University were selected between 2016 and 2020. No corticosteroid or immunosuppressive treatment was given in the 3 months prior to when tissue and blood samples were obtained. Serum IgG4 concentration was measured using the IgG4 latex-enhanced immunoturbidimetric kit (BYA13030, Byron Diagnostics, Shanghai). All the biopsy samples were excised from the major ophthalmic lesions. The biopsy sections of all the patients were reviewed by one experienced pathologist after ruling out all potential alternative diagnoses. A histopathological diagnosis of IgG4ROD was made in accordance with the diagnostic criteria proposed in 2014 [2]: ratio of IgG4+ cells to IgG+ cells of 40% or above, or more than 50 IgG4+ cells per high-power field (× 400).

### Histopathological data

Formalin-fixed and paraffin-embedded sections were stained with hematoxylin and eosin (HE). Slides of immunohistochemical staining of IgG4 and IgG (or CD138) were reviewed. IgG4+ plasma cells were counted in 5 nonoverlapping high-power fields (HPFs) with the highest density of stained positive cells at × 40 magnification. The average count was recorded. The average number of IgG+ plasma cells was also calculated in the fields where IgG4+ plasma cells were counted. Furthermore, HE-stained biopsy slides were morphologically screened for the presence of germinal centers (GCs), which was confirmed according to CD21 staining. The average number of GCs from 5 low-power fields (LPFs) with the densest concentration of lymphoid follicles at 5× magnification was counted.

### Clinical data

The electronic clinical records of all the patients were retrieved. All the patients underwent an extensive medical examination, and patients’ symptoms, laterality, and lesion locations were recorded. All 55 patients underwent axial and coronal magnetic resonance imaging (MRI) or computed tomography (CT) scans of the orbit, head, and neck before biopsy. Major ophthalmic lesions were classified by their anatomical location. Extraophthalmic lesions of the head and neck were determined by analyzing radiological images and performing a physical examination. Serum IgG4 concentration examinations were performed on peripheral blood from 31 patients at the time of diagnosis. The history of steroid treatment was recorded, and the treatment response and subsequent relapse after glucocorticoid regimens were reviewed. All the patients were followed up for more than 1 year.

### Statistical analysis

The analyses of the pathological counts of IgG4+ and IgG+ plasmacytes and GCs among the three groups were conducted using one-way ANOVA and LSD tests. The logarithm of the serum IgG4 level was introduced in a parametric test for the concentration following the lognormal distribution in IgG4ROD. The difference in the histopathological and serological findings between the groups with and without extraophthalmic involvement was analyzed using the unpaired Student’s *t* test. Linear regression analysis was performed to analyze the association between histopathological factors and serum IgG4 levels. Receiver operating characteristic (ROC) curve analysis was used to calculate the area under the curve (AUC) to identify diagnostic values of the counts of IgG4+ plasmacytes and serum IgG4 levels for IgG4ROD. Univariate and multivariate Cox regression analyses (enter method) were performed to identify the potential clinicopathological risk factors for relapse and their hazard ratios (HRs). Variables with *p* < 0.1 in the univariate Cox regression were included in the multivariate analysis. Kaplan–Meier survival curves and log-rank tests were performed to compare the effect of different grades of IgG4+ plasmacyte infiltration on IgG4ROD relapse. *p* < 0.05 was considered statistically significant. All statistical analyses were performed with SPSS statistics v.25 and R-project 4.1.2.

## Results

Fifty-five consecutive patients (30 men, 25 women; age range 15–72 years; mean age 54.5 years) were histopathologically diagnosed with IgG4ROD according to the diagnostic criteria. Fifty-one patients' IgG4 immunostaining sections demonstrated > 50 positive cells per HPF; although the IgG4-expressing plasmacytes per HPF were less than 50 in the 4 remaining patients, they were diagnosed with IgG4ROD based on the criterion of an IgG4+/IgG+ ratio > 40%. Bilateral lesions were found in 27 (49.1%) patients, and the rest exhibited unilateral lesions. The locations of the lesions were as follows: lacrimal gland, 43 (78.2%); extraocular muscle, 19 (34.5%); intraconal orbital soft tissue, 15 (27.3%); extraconal orbital soft tissue, 23 (41.8%); infraorbital nerve, 8 (14.5%); and conjunctiva, 1 (1.8%).

### Pathological findings in IgG4ROD lesions

All the biopsy samples were excised from the major ophthalmic lesions: 38 (69.1%) from the lacrimal gland, 6 (10.9%) from the extraocular muscle, 1 (1.8%) from the conjunctiva, and 10 (18.2%) from orbital soft tissues (extraconal and intraconal). All the IgG4ROD samples except one sample obtained from the conjunctiva were divided into three groups based on the anatomic location of the biopsy: the lacrimal gland group, the extraocular muscle group, and the orbital soft tissue group. Histopathologic examination of all the samples showed marked lymphocyte and plasmacyte infiltration. Monomorphous B cells and atypical lymphocytes was suspected focally in 6 cases and 2 cases, respectively. Detection of gene rearrangement was negative in these samples.

### GCs in IgG4ROD lesions

**Fig. 1:**
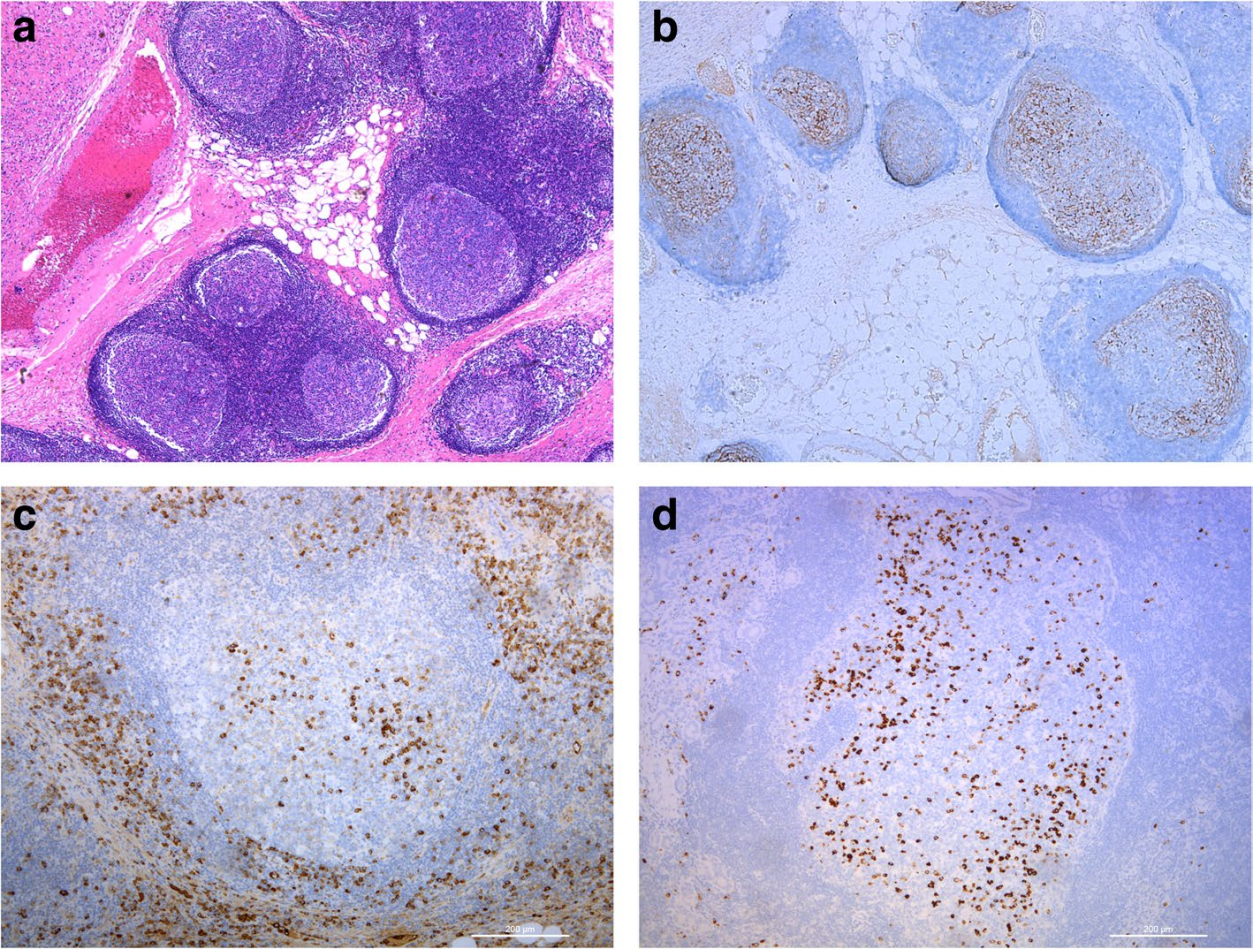
GCs and infiltrating IgG4+ plasma cells in lesions. **a**, **b** Lacrimal gland from patient no.7 showed abundant GCs that were confirmed by CD21 immunostaining (hematoxylin and eosin, **a**, **b**: × 50). **c** A GC was surrounded by numerous IgG4-positive cells and fibrosis. Only sparse IgG4+ plasma cells were distributed within the GC (× 100). **d** A majority of IgG4+ plasmacytes were confined to GCs (100×)

**Fig. 2:**
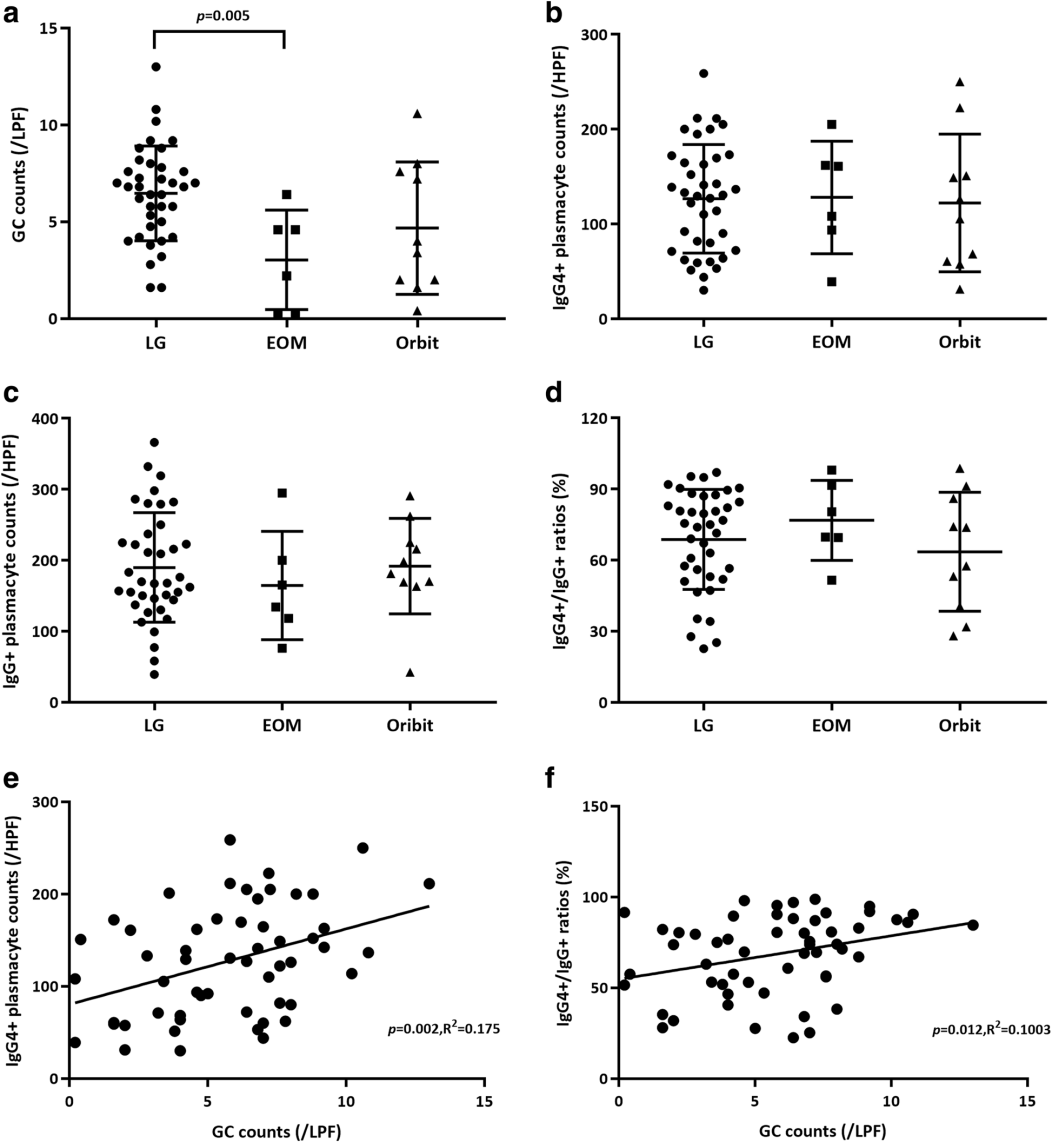
Quantity of pathological factors in lesions from different ophthalmic sites. **a** GC. **b**–**d** IgG4+ plasmacytes, IgG+ plasmacytes and their ratio. Correlation between the counts of GCs and infiltrating IgG4+ plasmacytes. **e** GC and IgG4+ plasmacyte. **f** GC and IgG4+/IgG+ ratio

GCs were observed in all the patients and were further confirmed by reviewing immunostaining for the follicular dendritic cell marker CD21 (Fig. 1a, b). The numbers of GCs in different lesions for the lacrimal gland, extraocular muscle, and orbital soft tissue groups were 6.47 ± 2.45, 3.03 ± 2.57, and 4.68 ± 3.42, respectively. A significant difference was found in the multiple comparison test among the three groups (*p* = 0.008, the lacrimal gland group vs. the extraocular muscle group, *p* = 0.005, Fig. 2a).

### IgG4+ plasma cells in IgG4ROD lesions

In our study, IgG4+ plasma cells were detected in the regions surrounding lymphoid follicles and were usually associated with fibrosis in the majority of our samples (Fig. 1c). In some cases, however, the IgG4+ plasma cells were mainly confined to GCs (Fig. 1d). The IgG4+ plasmacyte counts in the lacrimal gland, extraocular muscle, and orbital soft tissue groups were 126.6 ± 57.2, 128.1 ± 59.4, and 122.2 ± 72.6, respectively, and were not significantly different (*p* = 0.975, Fig. 2b). The IgG+ plasmacyte counts were also calculated, and those for the lacrimal gland, extraocular muscle, and orbital soft tissue groups were 190.8 ± 78.0, 164.6 ± 76.3, and 182.7 ± 58.0, respectively, and were not significantly different (*p* = 0.734, Fig. 2c). In addition, the IgG4+/IgG+ ratios in the lacrimal gland, extraocular muscle and orbital soft tissue groups were 69.1 ± 21.4%, 77.0 ± 16.7%, and 61.0 ± 25.3%, respectively, and were not significantly different (*p* = 0.487, Fig. 2d).

### Relationship between GC formation and plasmacyte infiltration

The pathological IgG4+ plasmacyte counts were positively correlated with the number of GCs in ophthalmic lesions (*p* = 0.002 < 0.05, *R*^2^ = 0.175, Fig. 2e). The IgG4+/IgG+ ratios were also slightly positively correlated with the GC counts in LPFs (*p* = 0.012, *R*^2^ = 0.1003, Fig. 2f). There was no correlation between the number of GCs and the pathological count of IgG+ plasmacytes (*p* = 0.167, *R*^2^ = 0.0299, figure not shown).

### Systemic conditions in patients with IgG4ROD

#### Serum IgG4 level in IgG4ROD patients

**Fig. 3:**
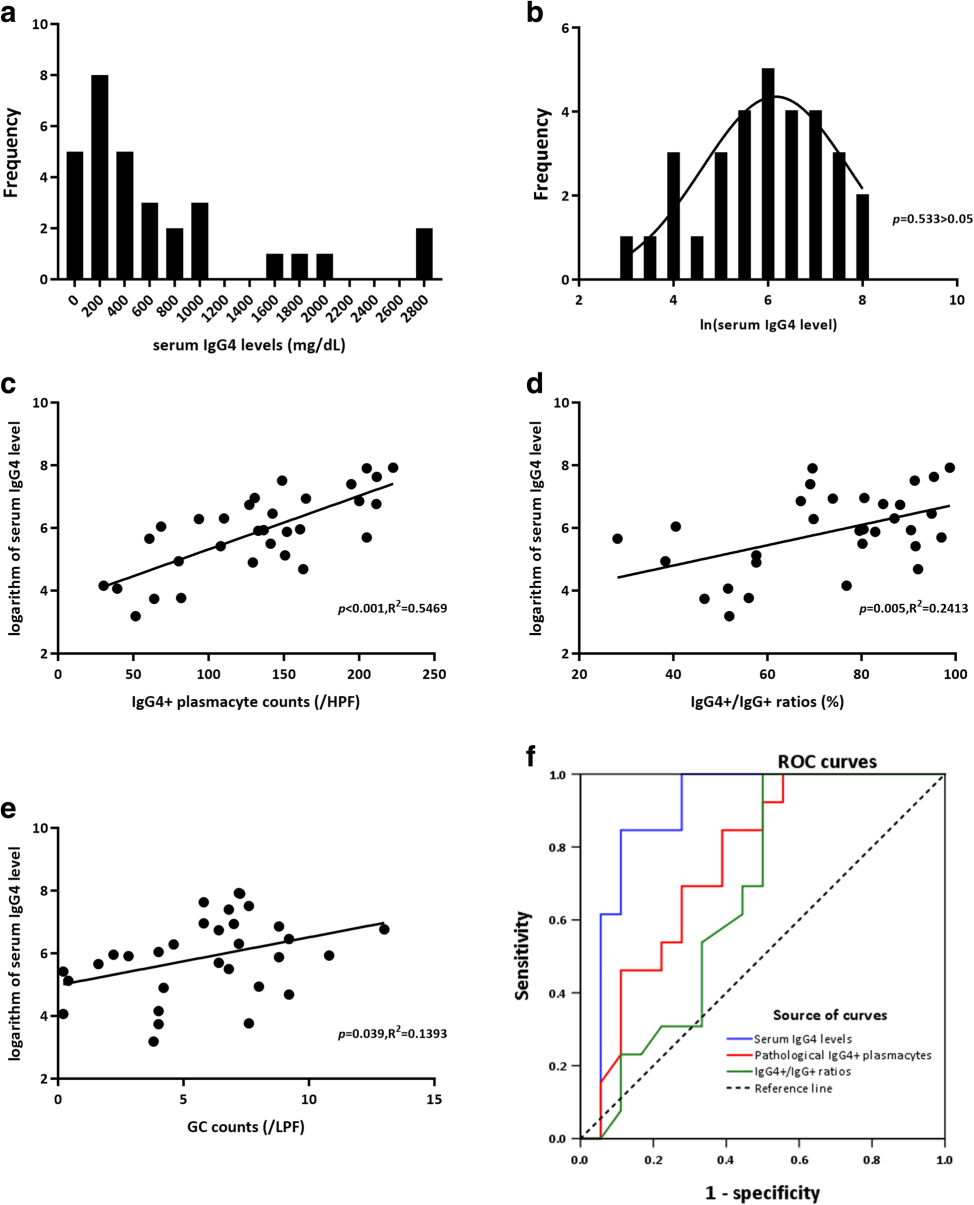
The distribution of the serum IgG4 level and its logarithm in patients with IgG4ROD. **a** Serum IgG4 level displayed a highly skewed distribution. **b** Logarithm of serum IgG4 level displayed a normal distribution. The correlation between the logarithm of the serum IgG4 level and pathological factors. **c** IgG4+ plasmacyte and ln (serum IgG4 level). **d** IgG4+/IgG+ ratio and ln (serum IgG4 level). **e** GC and ln (serum IgG4 level). Receiver operating characteristic curve (ROC) analysis. **f** ROC curves to evaluate the predictive performance of serum IgG4 level, IgG4+ plasmacyte, and IgG4+/IgG+ ratio for systemic involvement

The serum IgG4 concentration was greater than 135 mg/dL in 24 (77.4%) of the 31 IgG4ROD patients whose peripheral blood had been tested at diagnosis. The serum IgG4 levels are indicated with medians and quartiles, and the median concentration was 376 mg/dL (interquartile range 139 to 866 mg/dL). As shown, the frequency of the serum IgG4 level in the IgG4ROD patients displayed a highly skewed distribution (Fig. 3a). The logarithm of the serum IgG4 concentration exhibited a normal distribution (*p* = 0.533, Fig. 3b).

#### Relationship between serum IgG4 level and plasmacyte infiltration

The logarithm of the serum IgG4 level was included in the analysis of the association between the serum IgG4 concentration and the number of infiltrating plasmacytes. As shown, the counts of IgG4+ plasma cells and the logarithm of the serum IgG4+ level showed a strong and significantly positive correlation (*p* < 0.001, *R*^2^ = 0.5469, Fig. 3c). The IgG4+/IgG+ ratio and GC count were also positively correlated with the IgG4 concentration in the peripheral blood with a rather lower coefficient of determination (*p* < 0.05, Fig. 3d, e).

#### Extraophthalmic lesions of the head and neck

Sixteen (29.1%) of the 55 histopathologically diagnosed patients with IgG4ROD had extraophthalmic lesions of the head and neck. These lesions were observed on the pituitary gland in 2 patients, the parotid gland in 5 patients, the submandibular gland in 7 patients, the lymph nodes in 2 patients, the thyroid gland in 1 patient, the pterygopalatine fossa in 8 patients, and the cheek skin in 2 patients. Compared with those without multiorgan involvement in the head and neck, the patients with multiorgan involvement exhibited significantly higher serum IgG4 concentrations and pathological counts of infiltrating IgG4+ plasmacytes. These patients also presented a higher ratio of pathological IgG4+ plasmacytes to IgG+ plasmacytes and a higher rate of bilaterality (Table 1).

**Table 1:** Comparison of the clinicopathological findings in patients with and without extraophthalmic lesions of the head and neck

Clinicopathological factor	Extraophthalmic lesions of head and neck		*p* value
	With (***n*** = 16, 29.1%)	Without (***n*** = 39, 70.9%)	
Age of disease onset (years)	54.1 ± 10.7	51.8 ± 12.5	0.298
Sex (male, %)	51.3	62.5	0.448
Bilaterality (%)	75	38.5	0.014*
Lacrimal gland involvement (%)	87.5	74.4	0.284
ln (serum IgG4 level)	6.8 ± 0.7	5.2 ± 1.2	< 0.001*
GC (LPF)	6.7 ± 2.5	5.3 ± 2.9	0.099
Pathological IgG4+ plasmacyte (HPF)	161.9 ± 49.5	113.1 ± 58.1	0.005*
Pathological IgG+ plasmacyte (HPF)	201.2 ± 64.0	185.4 ± 77.9	0.476
Pathological IgG4+/IgG+ ratio (%)	82.8 ± 11.5	63.6 ± 22.5	0.002*

*ln* natural logarithm, *GC* germinal center, *p < 0.05

The performance of the serum IgG4 level and the pathological count of IgG4+ plasmacytes for predicting systemic involvement in patients with IgG4ROD was evaluated by ROC curve analysis. The AUCs of the serum IgG4 level, pathological IgG4+ plasmacyte count, and IgG4+/IgG+ ratio were 0.897 (95% CI: 0.777–1.000, *p* < 0.001), 0.759 (95% CI: 0.589–0.928, *p* = 0.015), and 0.669 (95% CI: 0.476-0.862, *p* = 0.114, Fig. 3f), respectively. The AUC using the serum IgG4 level (= 0.934) and the pathological count of IgG4+ plasmacytes (= 0.745) was moderately accurate for predicting systemic involvement in patients with IgG4ROD because both AUC measures fell between 0.7 and 0.9. The largest Youden index of 0.735 [(sensitivity−[1 − specificity] = 0.735)] suggested a cutoff value for the serum IgG4 level of 478.5 mg/dL. The sensitivity and specificity were 0.846 and 0.889, respectively. Regarding the pathological IgG4+ plasmacyte count, the largest Youden index (0.457) suggested a cutoff value of 130/HPF, which resulted in a sensitivity of 0.846 and specificity of 0.611.

### Treatment and IgG4ROD relapse

#### Treatment and relapse

Forty-six of the 55 patients received oral steroid regimens as their initial postoperative treatment. Oral glucocorticoids were given at an initial dose of 0.5–0.6 mg/kg/day for 4 weeks and were then tapered by 5 mg every week over the following 6–8 weeks. One patient who underwent steroid withdrawal during the initial glucocorticoid treatment was excluded from this part of the study. Low-dose steroids (5 mg/day of prednisolone) were maintained for at least 2 months after the initial glucocorticoid treatment to prevent relapse. Forty-one (91.1%) of the 45 patients who took glucocorticoids responded well, and clinical remission was not achieved in the remaining patients. Twenty-four (58.5%) of the 41 patients in whom remission was achieved suffered recurrence during the follow-up period. Thirteen (54.2%) of the 24 relapses occurred within 6 months following steroid treatment. As shown in Table 2, the patients with relapse had a higher baseline level of serum IgG4 and more infiltrating plasmacytes in lesions. More GCs were also observed in their samples. Once the disease recurred, a greater steroid dosage of 0.6–0.8 mg/kg/day or an addition of immunosuppressive agents to glucocorticoids was used to achieve remission again depending on expertise.

**Table 2:** Comparison of the clinicopathological findings in IgG4ROD patients with and without relapse after steroid treatment

Clinicopathological factor	Relapse after steroid treatment		*p* Value
	With (***n*** = 24, 58.5%)	Without (***n*** = 17, 41.5%)	
Age of disease onset (years)	54.7 ± 8.5	48.8 ± 16.3	0.135
Sex (male, %)	50.0	47.1	0.853
Bilaterality (%)	62.5	47.1	0.326
Lacrimal gland involvement (%)	79.2	76.5	0.837
ln (serum IgG4 level)	6.4 ± 0.99	5.0 ± 1.26	0.002*
GC (LPF)	7.2 ± 2.7	4.0 ± 2.4	< 0.001*
Pathological IgG4+ plasmacyte (HPF)	155.7 ± 51.2	94.6 ± 49.0	< 0.001*
Pathological IgG+ plasmacyte (HPF)	199.42 ± 58.5	167.0 ± 74.4	0.127
Pathological IgG4+/IgG+ ratio (%)	80.4 ± 16.2	59.9 ± 21.8	0.001*
Systemic involvement (head and neck, %)	45.8	23.5	0.144

*ln* natural logarithm, *GC* germinal center, *p < 0.05

#### Factors associated with relapse

**Fig. 4:**
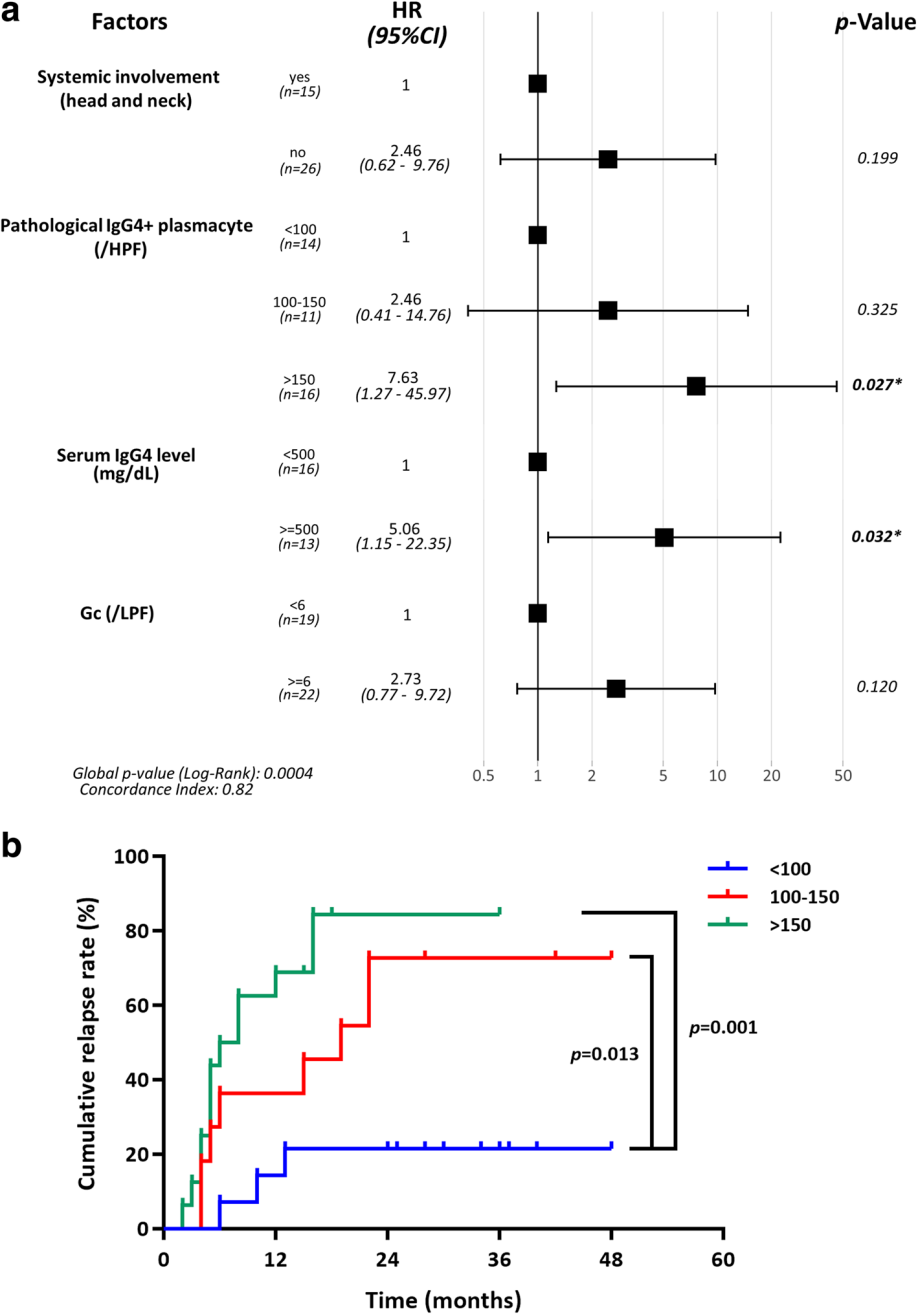
Forest plot of multivariate Cox regression analysis and curves of cumulative relapse rate for patients with IgG4ROD. **a** Forest plot of multivariate Cox regression analysis for IgG4-ROD relapse. Pathological IgG4+ plasmacyte count of > 150/HPF and elevated serum IgG4 level of > 500 mg/dL were risk factors associated with relapse after steroid treatment. **b** Kaplan–Meier curve of the cumulative relapse rate of IgG4ROD patients based on the grade of IgG4+ plasmacyte infiltration. The difference between curves of 100-150 and > 150 was not significant (*p* = 0.148 > 0.05). HR hazard ratio, CI confidence interval, GC germinal center. *p < 0.05

Through univariate Cox regression analysis, we identified that systemic involvement (head and neck), pathological GC and IgG4+ plasmacyte counts, and the level of serum IgG4 were associated with relapse (Table 3). Multivariate Cox regression analysis revealed that infiltrating IgG4+ plasmacytes > 150/HPF (HR 7.63, 95% CI 1.27–45.97, *p* = 0.027) and elevated serum IgG4 levels > 500 mg/dL (HR 5.06, 95% CI 1.15-22.35, *p* = 0.032) were risk factors for relapse (Fig. 4a). Kaplan–Meier curves of the cumulative relapse rate of IgG4ROD patients based on the numbers of pathological IgG4+ plasmacytes are plotted in Fig. 3b. The log-rank test proved that there was a significant difference between the cumulative relapse curves of the IgG4ROD patients with different degrees of IgG4+ plasmacyte infiltration. The patients with > 150/HPF pathological IgG4+ plasmacytes tended to have a higher cumulative relapse rate than those with fewer IgG4+ plasmacytes in lesions.

**Table 3:** Univariate Cox regression analysis for IgG4ROD relapse

Clinicopathological factor	Number (%)	HR (95% CI)	*p* value
Age of disease onset (years)	41 (100)	1.017 (0.985–1.050)	0.289
Sex			
Male	20 (48.8)	1	Ref
Female	21 (51.2)	0.767 (0.344–1.709)	0.516
Bilateral			
Yes	23 (56.1)	1	Ref
No	18 (43.9)	0.567 (0.247–1.301)	0.199
Lacrimal gland involvement			
Yes	32 (78.0)	1	Ref
No	9 (22.0)	0.956 (0.584–1.567)	0.859
Systemic involvement (head and neck)			
Yes	15 (36.6)	1	Ref
No	26 (63.4)	0.711 (0.476–1.064)	**0.097**^**#**^
GC (/LPF)			
< 6	19 (46.3)	1	Ref
≥ 6	22 (53.7)	4.073 (1.603–10.350)	**0.003**^**#**^
Pathological IgG4+ plasmacyte (/HPF)			
< 100	14 (34.1)	1	Ref
100–150	11 (26.9)	4.697 (1.242–17.758)	**0.023**^**#**^
> 150	16 (39.0)	8.701 (2.394–31.620)	**0.001**^**#**^
Serum IgG4 level (mg/dL)			
< 500	16 (39.0)	1	Ref
≥ 500	13 (31.7)	4.055 (1.541–10.676)	**0.005**^**#**^

*HR* hazard ratio, *CI* confidence interval, *Ref* reference, *GC* germinal center, ^#^*p* < 0.1

## Discussion

Histopathological features and immunohistochemical staining results provide supportive evidence for the diagnosis of IgG4RD. Although all conditions within the spectrum of IgG4RD are associated with lesions rich in IgG4+ plasma cells, a certain variability in the count exists across organs. Generally, lesions in exocrine glands such as the lacrimal glands tend to have strikingly more IgG4+ plasmacytes than organs such as the pancreas and retroperitoneum, with a predominance of fibrosis. We summarized the samples from lesions in the lacrimal glands, extraocular muscles, and orbital soft tissues. Lesions in the extraocular muscles and orbital soft tissues shared similar pathological features with those in the lacrimal glands [16]. Several ophthalmic lesions had no significant difference in the quantitative findings of IgG4 and IgG staining or their ratio. This result suggested that there was no significant variability in the cutoff number of IgG4+ and IgG+ plasmacytes across lesions in the different anatomical sites in IgG4ROD patients.

Even though an elevated serum IgG4 concentration is not sufficient to diagnose IgG4RD, its clinical interpretation has been established. There is convincing evidence that the serum level of IgG4 in patients with multiple organ involvement is higher and correlated with disease activity [17]. In our study, the IgG4 concentration distribution skewed to the right. To improve the efficiency of the analysis, we performed a parameter test after certifying that the baseline serum IgG4 level in the IgG4ROD patients showed a logarithmic normal distribution. The serum IgG4 level of patients with a pathological diagnosis of IgG4RD from previous studies also followed a logarithmic normal distribution (*p* = 0.363, *p* = 0.940 and *p* = 0.236, respectively) [8, 16, 18]. Performing a parameter test makes the positive correlation between the quantity of pathological factors and the serum IgG4 concentration more convincing. A higher coefficient of determination was found for IgG4 staining than for IgG staining. It is possible that the counts of IgG+ plasma cells in fields were much more likely to reach saturation, which diluted their relevance to serum IgG4 levels. Similar results were found for other IgG+-related factors. Our study was the first to analyze the relationship between IgG4 levels in serum and IgG4+ plasmacytes in lesions. Considering the clinical application of serum IgG4 levels, this result provided evidence for the potential application of IgG4+ plasmacyte counts in the management of IgG4ROD.

We investigated the potential value of the count of IgG4+ plasmacytes to identify systemic involvement. Because extraorbital imaging was not performed in most patients, we chose extraophthalmic lesions of the head and neck to represent systemic conditions. It has been reported that the head and neck region is the second most common site for IgG4RD after the pancreas [19]. The patients without any extraophthalmic symptoms or lesions of the head and neck were regarded as having no systemic involvement. Lesions in the pterygopalatine fossa were regarded as extraophthalmic manifestations because we noticed that such lesions in 2 cases were isolated [14, 20]. Our results showed that the serum IgG4 level and the rate of bilaterality were higher in patients with systemic involvement. This result was consistent with that of a previous report [21]. More IgG4+ plasmacytes and a higher IgG4+/IgG+ ratio were also observed in these patient sections. The AUC using the serum IgG4 level was larger than that using the pathological IgG4+ plasmacyte count. For the identification of systemic lesions, a serum IgG4 cutoff value of 478.5 mg/dL had the same sensitivity of 0.846 and a higher specificity of 0.889 than a cutoff value of 130/HPF of pathological IgG4+ plasmacytes with a specificity of 0.661. This implies that compared with the serum IgG4 level, the quantity of pathological IgG4+ plasmacytes may have a relatively weaker efficiency for indicating systemic involvement in IgG4ROD patients. This result may be able to apply to other subtypes of IgG4RD with similarly numerous infiltrating IgG4+ plasmacytes.

Apart from the function in identifying systemic involvement, we also investigated the role of IgG4+ plasmacyte-related pathological findings in predicting relapse. Consistent with a previous study, the patients with elevated pretreatment levels of serum IgG4 were more likely to experience relapse after steroid treatment. The patients with relapse also tended to have more infiltrating IgG4+ plasmacytes and more GCs in lesions. Multivariate Cox regression analysis indicated that a > 150/HPF IgG4+ plasmacyte count and a > 500 mg/dL serum IgG4 level were risk factors for relapse. The practical value and quantity of pathological IgG4+ plasmacytes corresponded to the serum IgG4 concentration in patients with IgG4ROD.

The GC, the discrete lymphoid structure where high affinity B cells are selected and differentiate into plasma cells, is responsible for the generation of high-affinity IgG antibodies [22]. Compared to lesions in other ophthalmic sites, lesions in the lacrimal glands had more GCs. We found a positive correlation between the counts of GCs and IgG4+ plasmacytes with a low coefficient of determination. The positive correlation of the number of GCs with serum IgG4 levels and IgG4+ plasmacyte counts suggested that the development of IgG4 antibodies and IgG4+ plasmacytes may be associated with GCs. More GCs were observed in patients with relapse, but it was not the key risk factor for relapse in our study. This result indicates its potential value in clinical practice.

Taken together, this study demonstrated that the serum IgG4 level, which has been reported in previous studies, is of practical value in identifying systemic involvement and predicting relapse. The pathological IgG4+ plasmacyte count reflects the inflammation in localized lesions and is associated with local recurrence. It can also be used to identify systemic conditions with a lower specificity compared to that for the serum IgG4 level. The serum IgG4 level can be influenced by previous treatment, but baseline counts of pathological IgG4+ plasmacytes are always stable. This objective advantage of the pathological IgG4+ plasmacyte count over the serum IgG4 level lends itself to the management of IgG4ROD.

There were some limitations in this study. First, the subjects were limited to patients who were diagnosed pathologically by only one pathologist. We sent the questionable samples to Fudan University Shanghai Cancer Center for pathology consultation to reduce possible bias. Second, systemic imaging examinations were not performed to evaluate extraophthalmic involvement, and the serum IgG4 concentration was tested only in some patients. Due to the lack of sufficient information on systemic conditions, our results on systemic involvement might not be accurate. Third, some patients were referred to us after receiving oral or intravenous steroid treatment and experiencing recurrence elsewhere. Low-dose oral corticosteroid was only maintained long term in several patients with severe systemic conditions. This could influence the analysis of response to steroids and relapse.

In conclusion, our results suggest that various lesions from different ophthalmic sites provide similar quantitative results regarding IgG4 and IgG staining in patients with IgG4ROD. The number and ratio of IgG4+ plasmacytes are positively correlated with the serum IgG4 level. Both higher serum IgG4 levels and more infiltrating IgG4+ plasmacytes are associated with systemic lesions and relapse. The pathological IgG4+ plasmacyte count may be of practical value in the management of IgG4ROD.

## Data Availability

The datasets used and/or analyzed during the current study are available from the corresponding author on reasonable request.

## Abbreviations

IgG: Immunoglobulin G
IgG4: Immunoglobulin G4
IgG4ROD: IgG4-related ophthalmic disease
IgG4RD: IgG4-related disease
GC: Germinal center
HPF: High-power field
LPF: Low-power field
LG: Lacrimal gland
EOM: Extraocular muscle
CT: Computed tomography
MRI: Magnetic resonance imaging
HR: Hazard ratio
SD: Standard
CI: Confidence interval
Ref: Reference
ROC: Receiver operating characteristic
AUC: Area under the curve
ln: Natural logarithm
